# An analysis of atmospheric water vapor variations over a complex agricultural region using airborne imaging spectrometry

**DOI:** 10.1371/journal.pone.0226014

**Published:** 2019-12-06

**Authors:** Sarah W. Shivers, Dar A. Roberts, Joseph P. McFadden, Christina Tague

**Affiliations:** 1 Department of Geography, University of California, Santa Barbara, California, United States of America; 2 Bren School of Environmental Science & Management, University of California, Santa Barbara, California, United States of America; George Mason University, UNITED STATES

## Abstract

Understanding atmospheric water vapor patterns can inform regional understanding of water use, climate patterns and hydrologic processes. This research uses Airborne Visible Infrared Imaging Spectrometer (AVIRIS) reflectance and water vapor imagery to investigate spatial patterns of water vapor in California’s Central Valley on a June date in 2013, and 2015, and relates these patterns to surface characteristics and atmospheric properties. We analyze water vapor imagery at two scales: regional and agricultural field, to examine how the slope, intercept, and trajectory of water vapor interact with the landscape in a highly diverse and complex agricultural setting. At the field scale, we found significant quadratic relationships between water vapor slope and wind magnitude in both years (p<0.001). Results showed a positive correlation between crop water use and the frequency with which crops showed directional agreement between wind and water vapor (r = 0.23). At the regional scale, we found patterns of water vapor that indicate advection of moisture across the scene. Water vapor slope was inversely correlated to field size with correlations of -0.37, and -0.28 for 2013 and 2015. No correlation was found between green vegetation fraction and vapor slope (r = 0.001 in 2013, r = 0.02 in 2015), but a weak correlation was found for the intercept (r = 0.11 in 2013, r = 0.26 in 2015). These results lead us to conclude that accumulation of water vapor above fields in these scenes is observable with AVIRIS-derived water vapor imagery whereas advection at the field level was inconsistent. Based on these results, we identify new opportunities to use and apply water vapor imagery to advance our understanding of hydro-climatic patterns and applied agricultural water use.

## Introduction

Boundary layer atmospheric water vapor is a critical element of climate, an indicator of land surface hydrologic processes, and a potent greenhouse gas [1,2]. As such, analysis of vapor patterns at a fine spatial scale can inform climate and plant water use studies [3,4]. Imaging spectrometers such as NASA’s Airborne Visible Infrared Imaging Spectrometer (AVIRIS) measure reflected radiance at fine spatial and spectral resolution, and in so doing provide measurements of column water vapor as well as a highly detailed reflected signal from the land surface below [5–7]. These two spatially corresponding products allow us to uniquely observe surface processes and characteristics as they relate to the atmospheric patterns above them. As such, this research proposes to leverage water vapor and reflectance imagery to observe and assess spatial patterns of water vapor in the Central Valley of California to evaluate the assets and limitations of this dataset for evaluation of agricultural water use.

Observation and evaluation of water vapor over agricultural fields in California’s Central Valley have substantial value for water resource management, irrigation assessments, and regional climate patterns. The Central Valley contains one of the world’s largest contiguous areas of high irrigation density [8] with more than 3.6 million irrigated hectares of farmland [9] that use over 80% of the state’s managed water supply [10]. Worldwide, arid and semi-arid regions such as the Central Valley see upwards of ninety percent of precipitated water returned back to the atmosphere via evapotranspiration (ET) [11]. In the Central Valley, where precipitation is low and managed water inputs are extreme, annual ET exceeds precipitation by about 60% [12,13]. These extreme irrigation inputs, therefore, significantly modify the spatial and temporal distribution of hydrologic flows across the region by transforming liquid water resources (from the surface or ground) into transpired atmospheric water vapor [14] that can be transported and distributed as rainfall elsewhere [13]. Further, as local atmospheric water vapor is intensified by ET, it is indicative of agricultural water inputs and crop functioning throughout the region.

Imaging spectrometers such as AVIRIS quantify column water vapor using several water absorption features across the infrared portion of the electromagnetic spectrum as part of the reflectance retrieval process [15,5,16]. Atmospheric water vapor absorption features occur at 0.94, 1.14, 1.38 and 1.88 μm, and the relative depth of these features can be used to derive atmospheric water content [17]. Hyperspectral imagery is uniquely suited to estimate water vapor because its high spectral resolution captures water absorption features that multi-spectral sensors, such as Landsat, are designed to avoid. In addition, the fine spatial resolution of the retrievals (~18 m) enable observation of water vapor patterns that are obscured in spatially coarser imagery, such as MODIS (1 km) or GPS meteorology [18], which estimates column water vapor based on delays in the signal between GPS satellites and a receiver.

**Fig 1:**
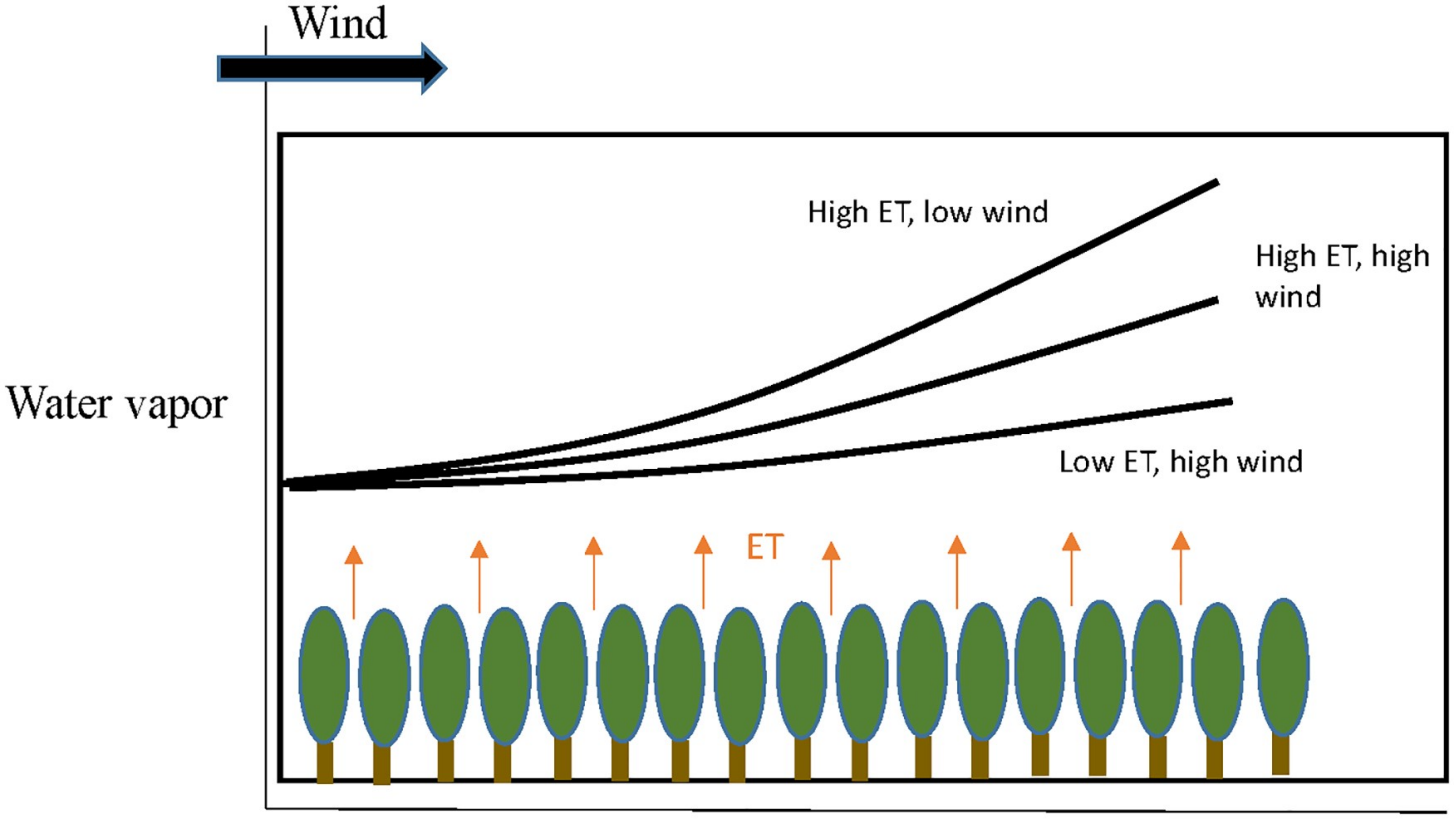
Conceptual schematic of columnar water vapor as it is affected by ET rates and wind, modified from Ogunjemiyo et al. [4].

While water vapor imagery is produced as a byproduct of most visible to shortwave infrared (VSWIR) reflectance retrievals, few analyses have been conducted with this rich dataset, leaving many questions as to the utility of these data unanswered. A notable exception is the work of Ogunjemiyo et al. [4] who studied water vapor over poplar plantations in Washington State to assess the feasibility of using AVIRIS-retrieved column water vapor as a tool to study plant ET. That study proposed a conceptual model of water vapor and its relationship to the surface (Fig 1), hypothesizing that plants with higher rates of transpiration will produce more water vapor, which will advect downwind and accumulate to a level detectable in the imagery, and that crops with higher water use rates will have steeper water vapor slopes, modified by wind speeds. Ogunjemiyo et al. [4] found that the patterns and magnitude of retrieved water vapor in their study areas were consistent with wind direction and reasonable transpiration rates for poplars with unlimited access to water, concluding that AVIRIS water vapor is sensitive to ET under certain boundary layer conditions.

Here, we build upon the work of Ogunjemiyo et al. [4] to further evaluate if AVIRIS is sensitive to field scale ET by testing a series of specific hypotheses to investigate how AVIRIS estimates of water vapor vary with the surface properties and atmospheric conditions that might be expected to influence water vapor in a complex agricultural environment (Table 1). Relating water vapor patterns to crop water use will be more challenging in the Central Valley of California than the more simplified landscape in Ogunjemiyo et al [4], which consisted of large fields of a single, well-watered crop. Crop water use and water vapor patterns are likely to be more complex in the Central Valley because this landscape includes a more variable arrangement of agricultural fields with different crop types, land management, irrigation practices, field sizes, and vegetation cover fractions. Table 2 summarizes the biological and meteorological mechanisms that might influence the relationship between land surface characteristics (e.g crop type and arrangement) and atmospheric water vapor. These mechanisms extend the simple conceptual model of plant-atmosphere interactions given a well-watered homogeneous crop (Fig 1). To examine water vapor patterns in this more complex landscape, we analyze two AVIRIS/MASTER data sets acquired in the Central Valley, California as part of the HysPIRI Airborne Campaign, one in 2013, the other 2015. Both data sets were acquired in June, prior to solar noon over heavily irrigated fields when rates of evapotranspiration should be high and water vapor still strongly coupled to the surface. A third data set was available from June, 2014, however, that data set was acquired much later in the day under less ideal conditions. Initial inspection of this image showed little or no relationships between water vapor and surface patterns. In this case water vapor may be dominated by upper atmospheric mechanisms (clouds, larger scale transport) and decoupled from the surface. Here we choose to utilize only images where coupling with the surface is detectible.

**Table 1:** Hypotheses to be tested in this study.

	Hypotheses
	Scene Level
**A**	Water vapor imagery will show increasing water vapor (advected moisture) in the downwind direction.
	Field Level
**B**	Wind direction and water vapor trajectories will closely align
**C**	The slope of the water vapor trajectory will vary with wind speed, peaking at intermediate wind speeds. At light or inconsistent winds, gradients will not be observed but water vapor will build up over fields. At high winds, the slope of the water vapor gradient will shallow.
**D**	The slope of the water vapor gradient will increase with field size.
**E**	The slope and intercept of the water vapor gradient will positively correlate with green vegetation fraction within vegetated fields.
**F**	High water demand crops will have steeper gradients.
**G**	Crops with lower leaf temperatures will produce steeper gradients

**Table 2:** Canopy-level and field level factors affecting plant/atmosphere interactions [22].

Factor	Effect
Crop Type	
Aerodynamic roughness	Aerodynamically rough vegetation is more coupled to the atmosphere because it creates turbulence which transfers heat more readily from the surface to the atmosphere. Short, smooth surfaces are less coupled than taller, rougher surfaces.
Vapor pressure deficit (VPD)	Transpiration increases with increasing VPD up to a certain point at which stomata close and transpiration declines. The tipping point differs among species [23].
Canopy conductance	The rate at which water vapor exits plant stomata will moderate the concentration of vapor above the canopy, and this varies by plant species.
Field Size	
Edge effects	The size of the field affects the proportion of land that exists at the boundary with another field. Smaller fields are more susceptible to edge effects of neighboring fields than larger fields.
Climatic	
Variability	A larger and more dynamic study scene will experience greater ranges in wind speed and direction and vapor pressure deficit than a more localized study area.

We analyze water vapor at two scales–field, and scene. Our hypotheses (Table 1) are designed to explore whether consistent (in time and space) relationships between imagery, atmospheric conditions, and surface properties may be derived from hyperspectral imagery in a complex agricultural landscape and at which scales. We assume active ET is occurring given the date (peak growth) and time (prior to solar noon) of the acquisition and heavy use of irrigation in the Central Valley. Prior work demonstrating an inverse relationship between green vegetation cover and Land Surface Temperature (LST) for these data sets [19] further supports an assumption of active ET. If ET is occurring, evapotranspired moisture should advect downwind. At the regional or scene scale, water vapor concentration should increase downwind due to moisture advection. For example, if the wind is blowing from the North, fields in the southern part of the study area should show more water vapor than fields in the northern part of the study area (Hypothesis A). At the field scale, gradients (from low to high moisture) should form that follow wind direction (Hypothesis B). The relationship between water vapor slope and wind should be quadratic with relatively high or low winds creating water vapor gradients less steep than winds that are of an “intermediate” magnitude. Only at intermediate winds, will a strong linear trend be observed. Light and/or inconsistent winds will not produce any gradients while higher winds will move water vapor at a faster rate, leading to shallower gradients (Hypothesis C: Fig 1). Field conditions (e.g. GV fraction and field size) should impact relationships between water vapor and wind. Thus we also investigate how these properties impact our evaluation of Hypothesis B and C. Field size, which will impact fetch, should impact the gradient (D). Fields below some critical threshold in size would not contribute enough water vapor to generate a detectable gradient whereas larger field should generate steeper gradients.

The gradient should also vary with vegetation cover (Hypothesis E). Positive correlations would indicate that fields with more vegetation are adding more moisture to the air than less vegetated fields. Similarly, fields with higher water-demand crops should have more pronounced gradients (Hypothesis F). Hypotheses A-F assume that vegetation is transpiring. As noted above the June data and the widespread use of irrigation in the Central Valley means that this will often be the case. We do not have direct measurements of crop ET. However we utilize Land Surface Temperature (LST, leaf temperatures) as an indicator of plant activity. Fields with lower LST are assumed to consist of healthier and less stressed plants than those with higher LST because plants that have adequate water will transpire and cool themselves [20,21]. Thus fields with lower leaf temperatures should have steeper water slopes (Hypothesis G).

Results will identify opportunities and limitations of using water vapor imagery to study ET at the ground surface in this important agricultural region.

## Methods

### Study area

**Fig 2:**
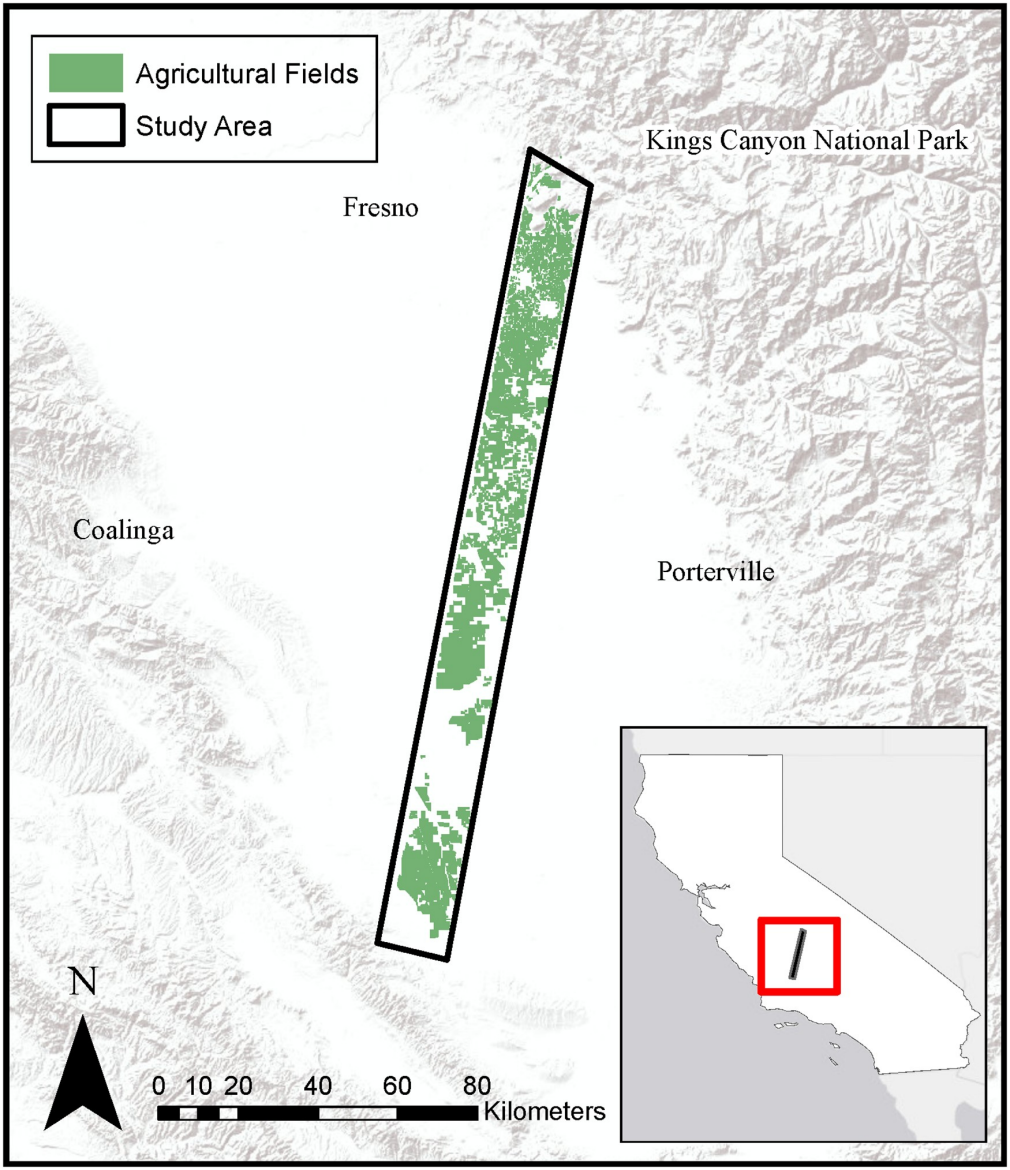
Study area within California’s Central Valley. Basemap obtained from Esri ArcGIS. Sources: Esri, DeLorme, HERE, MapmyIndia.

This research focuses on a transect of the California Central Valley that was flown as part of the Hyperspectral Thermal Imager (HyspIRI) Airborne Preparatory Campaign (Fig 2). The study area includes portions of Fresno, Tulare, Kern, and Kings Counties, three of the top four leading agricultural counties in California [24]. The study area is part of the Tulare Lake Hydrologic Region, which comprises the southern third of the Central Valley and is the largest agricultural region in California with about 1.2 million irrigated hectares out of its 4.4 million hectares total [25]. The region is prosperous for agriculture partially because of its long growing season, with moist winters often blanketed by fog and dry summers [25]. Tulare Lake is the driest region of the Central Valley, receiving an average of less than 25.4 cm of precipitation a year [25, 26].

### Data

#### Radiance and reflectance imagery

The Surface Biology and Geology (SBG) mission was identified in the 2017 Decadal Survey as a designated program element prioritized for development as a means to enhance our ability to monitor ecosystems, natural hazards, and land use over time [27]. The proposed sensor will capture a large number of spectral bands in the visible and shortwave infrared at a 30 m resolution, as well as multiple bands in the thermal infrared (TIR) at a 60 m resolution. The HyspIRI Airborne Campaign (HAC) offers the potential of evaluating many of the capabilities of SBG in advance of the mission. It flew the AVIRIS and MODIS-ASTER Simulator (MASTER) instruments throughout California seasonally from 2013 to 2017 at an altitude of 20 km. AVIRIS is a 224 band imaging spectrometer that captures wavelengths from 350 nm—2500 nm at approximately 10 nm increments [6]. MASTER captures 8 bands in the TIR region from 4–12 μm [28]. NASA’s Jet Propulsion Lab (JPL) preprocessed the imagery and produced orthorectified, high spectral resolution radiance and atmospherically-corrected reflectance imagery from AVIRIS at an 18 m spatial resolution and a LST product from MASTER at a 36 m spatial resolution. AVIRIS imagery was resampled to 36 m by pixel aggregation for consistency with LST imagery. Two dates of imagery from HAC were analyzed: June 6, 2013, and June 2, 2015 collected at 18:25, 21:41, and 18:59 UTC, respectively.

#### Water vapor imagery

Pixel-level water vapor estimates were calculated from the radiance imagery using Atmospheric CORrection Now (ACORN) 6.0 atmospheric correction software [29]. ACORN 6 models atmospheric gas absorptions and scattering effects using nonlinear least-squares spectral fitting with look-up tables of water column densities generated from MODTRAN 4 radiative transfer runs [30,31]. The result is pixel-level water vapor and liquid water estimates over the entire study scene. ACORN 6 was run in mode 1.5 for atmospheric correction of hyperspectral data. ACORN 6, mode 1.5 inverts AVIRIS radiance to solve for surface reflectance while simultaneously estimating column water vapor. Chosen parameters include the use of both 0.94 μm and 1.14 μm to derive water vapor, a mid-latitude summer model, an average surface elevation of 100 meters, automated estimate of visibility and artifact suppression.

#### Surface and field properties

The studied agricultural landscape was characterized using both the reflectance imagery and a geographic information system (GIS) layer of field data. Multiple Endmember Spectra Mixture Analysis (MESMA) [32] was applied to AVIRIS reflectance imagery to estimate pixel-level fractions of green vegetation (GV), soil, and non-photosynthetic vegetation (NPV). Spectral Mixture Analysis is a technique that estimates fractional cover of key components within a scene as linear combinations of the pure (unmixed) spectra of those components, called endmembers [33]. MESMA is a variant of standard SMA, in that it allows the number and types of endmembers to vary on a per pixel basis, thus accounting for within class endmember variability [32]. Endmembers can be derived from field-based spectral libraries, laboratory measurements or from imagery. In this study, spectral libraries for NPV, GV and Soil were derived from AVIRIS imagery from densely vegetated (GV), senesced (NPV) and bare soil fields (Soil). MESMA was run on each of the image dates using one spectral library of 40 image-selected endmembers (22 GV, 8 NPV, 10 Soil) that were collected from all dates. In addition to cover fractions, we obtained field size and crop species data. This information was obtained from GIS crop data layers provided by Tulare, Kings, Kern, and Fresno Counties. Field boundaries and crop information are gathered as part of a California’s required registration and permitting of agricultural fields that use pesticides. Field sizes ranged from <100 m^2^ to 2.7 km^2^. Using the MESMA results and the crop map, which delineates field boundaries, a mean estimate of GV, Soil, and NPV was calculated for each field.

#### Wind patterns

**Fig 3:**
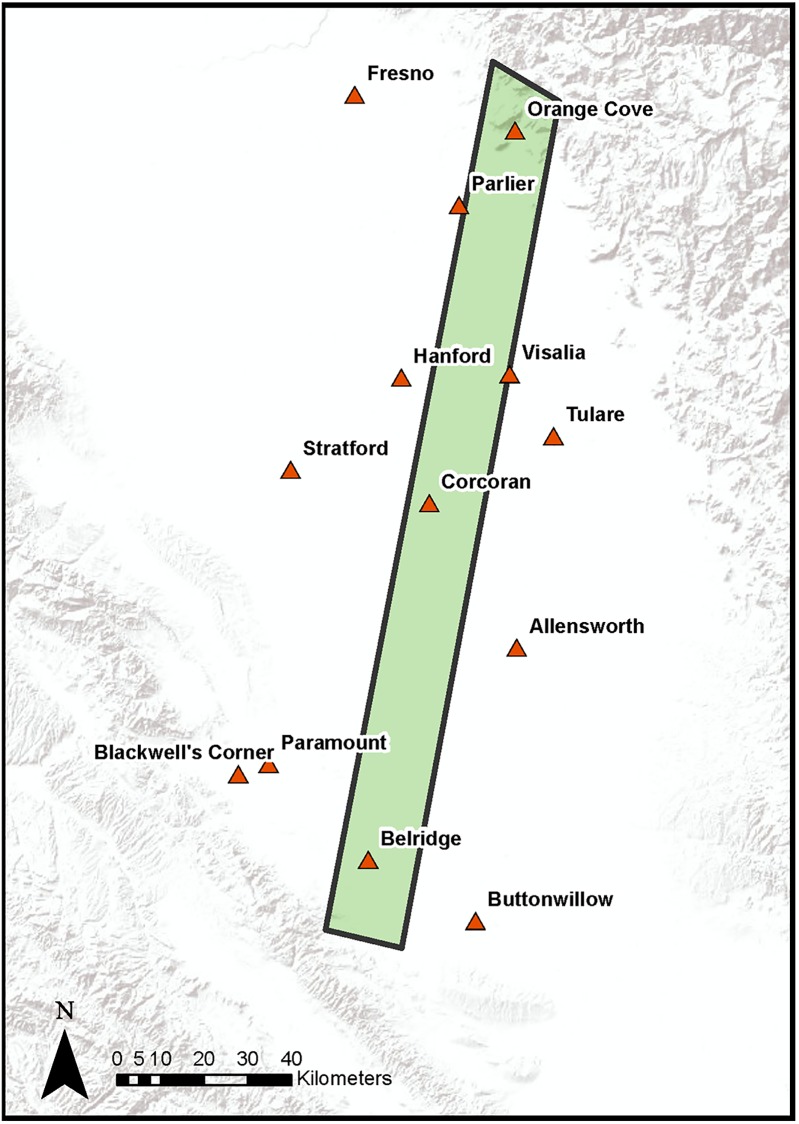
Meteorological stations used for wind speed and direction interpolation. Thirteen meteorological stations are shown in juxtaposition with the study site in light green. Basemap obtained from Esri ArcGIS. Sources: Esri, DeLorme, HERE, MapmyIndia.

Advected water vapor should vary locally depending on wind speed and direction. Therefore, we interpolated a map of wind over the study area during the study times and dates in order to create an estimate of wind activity against which to compare water vapor patterns. To calculate wind speed and wind direction, we relied on weather station data from 13 meteorological stations in or within close proximity to the study area (Fig 3). Weather stations used in the analysis are managed by various sources including government agencies, private firms, and educational institutions. Meteorological data were downloaded from the MesoWest and California Irrigation Management Information System (CIMIS) networks. For each station, the wind speed and direction that most closely matched the flight time for each date was recorded. All observations were within 30 minutes of the flight time. Using the 13 data points for wind magnitude and direction, data were spatially interpolated across the study area using an inverse weighted distance (IDW) formula. IDW relies on the idea that each estimated data point will be influenced by the known data surrounding it, and this influence will diminish with distance. IDW has been widely used in climatic studies for interpolating data such as temperature, rainfall, and wind [34] and is computationally efficient [35], but has been found to have low accuracy when data are sparse or unevenly distributed [36]. As our study area has low relief and stations are somewhat evenly disbursed, we felt IDW would be an appropriate and efficient method for wind interpolation. At the extreme north and south ends of the line, elevations range from 900 m (south) to 600 m (north). However, along the densely vegetated portions of the line, elevations only range from 53 to 120 m with no significant relief. The result is a pixel-level estimation of wind speed and wind direction across the study area for each of the study dates.

#### Field-level water vapor trends

**Fig 4:**
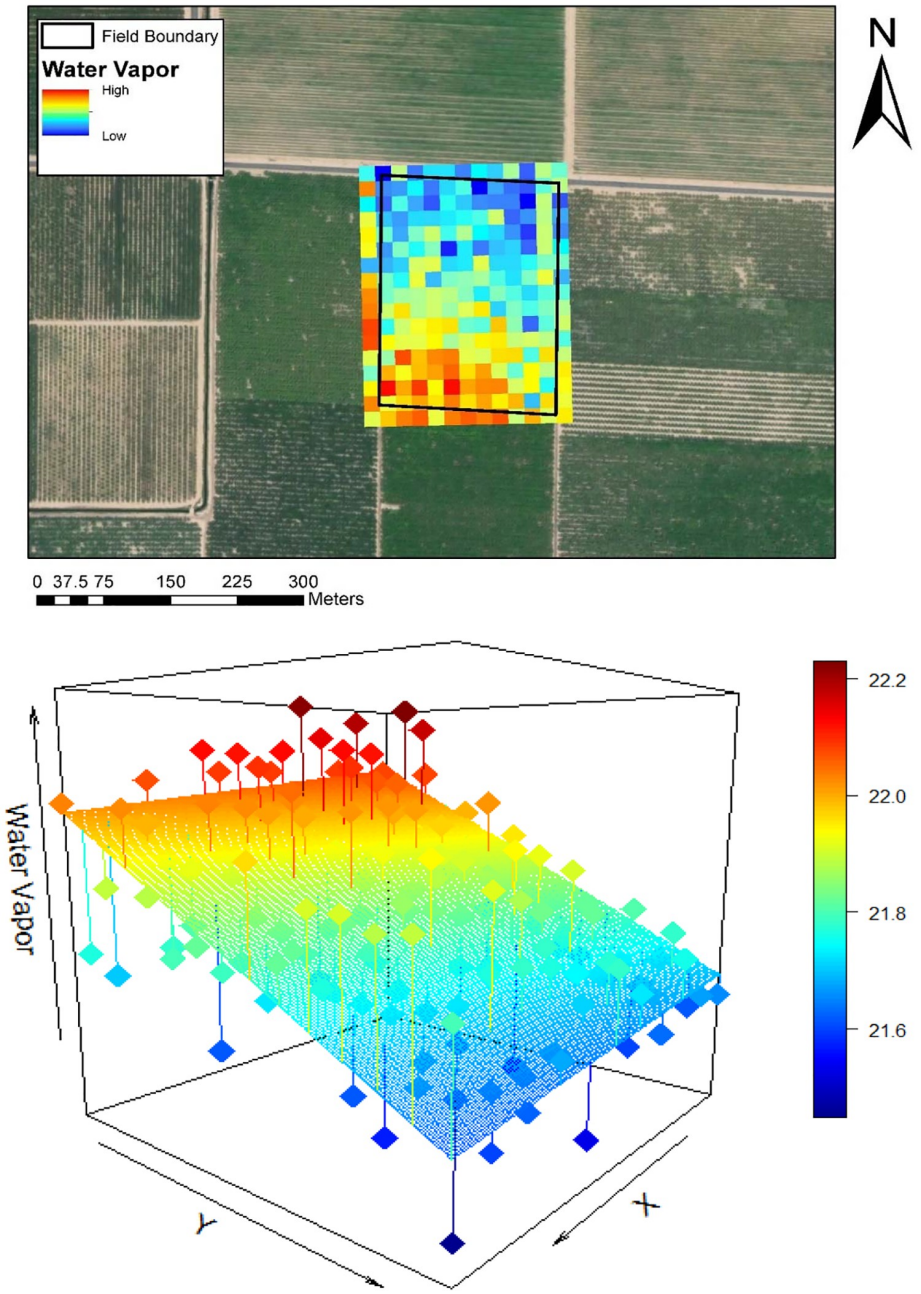
Water vapor trend occurring over an example field. The top graphic shows pixel-level water vapor measurements from 2013 from AVIRIS above polygon “152351”, a field of nectarines. The bottom plot illustrates the same water vapor measurements, fitted by a linear trend surface. Column water vapor values reported in millimeters. Slope = 0.99 and r^2^ = 0.61. Basemap obtained from Esri ArcGIS. Sources: Esri, DigitalGlobe, GeoEye, i-cubed, USDA FSA, USGS, AEX, Getmapping, Aerogrid, IGN, IGP, swisstopo, and the GIS User Community.

Water vapor analysis was performed at the field scale to reduce the impact of pixel-level variation and allow for sufficient fetch to retrieve water vapor gradients from individual fields. Fields were defined from the GIS crop data layers. To evaluate whether a gradient was present over a field, pixel-based estimates of column water vapor were derived for each field, then a 3D linear trend surface was fitted to the water vapor data. We acknowledge that non-linear trends are possible, but restrict our analysis here to only those fields were the linear trend was significant. This was done for every field defined by the GIS drop data for 2013 and 2015, (n = 2599 for 2013; n = 3197 for 2015, where n is the number of fields). Using the fitted surface, an intercept, slope, and trajectory were calculated for each field to explore the magnitude, rate of change, and directionality of the water vapor above it. The slope acts as a measure of moisture advection as a factor of wind at the field-level. The intercept is an important measure of water vapor at both the field and scene levels. At the scale of an individual field, the intercept quantifies the build-up of moisture over a field, while at the scale of the entire study site, the spatial pattern of intercepts highlight advection of moisture across the scene. The trajectory (aspect of the best-fit surface) is equivalent to the azimuth of the water vapor trend at the field level. To assess the statistical significance of the modeled, fitted surface, r-squared and p-values were also calculated. Only fields that had statistically significant linear trends (p<0.05) were analyzed (84% in 2013, 92% in 2015). The water vapor occurring above an example field and its corresponding fitted plane are shown in Fig 4.

### Analysis

#### Water vapor across-scene analysis

We tested Hypothesis A by examining patterns of water vapor intercepts against prevailing wind direction. We evaluated this hypothesis in each year by mapping out intercepts in the study area and qualitatively assessing their relationship to the calculated wind direction.

#### Water vapor field-level analysis

At the field level, we analyzed gradients of water vapor as they vary over agricultural fields as conceptualized in Fig 1 and as explained through Hypotheses B through F in Section 1. As such, we tested Hypotheses B and C by evaluating the relationship between wind speed and direction with the slope of water vapor. Even if pixel or scene-level trends were not identified in an image, we included both dates of imagery in the field-level analysis as we hypothesize that trends may occur at variable scales such that a trend can be significant at one scale but non-significant at a different scale. The trends of water vapor above fields will be a factor of both wind speed and direction. To test hypothesis B and C we plotted wind magnitude against water vapor slope in each year. We calculated the difference between the estimated wind direction and the trajectory of the water vapor above each field as the directional difference. For those fields that had directional differences of less than 30° and a statistically significant slope of vapor, we analyzed their characteristics such as crop type and GV fraction to understand what types of fields our set of hypotheses holds for.

Second, we tested the impact of field size on water vapor slope in fields of >50% GV to examine Hypothesis D. We plotted field size against water vapor gradient.

Third, we examined the relationship between GV fraction and water vapor slope in order to test hypothesis E. We separated fields into groups of similar field size to control for the impact of field size and then estimated the correlation between green vegetation cover and water vapor slope and intercept within each of those groups. We compared water vapor slope and intercept in fields with lower vegetation cover (<50% GV) with fields containing a majority GV fraction (>50% GV). We used a 50% GV threshold as was set in Shivers et al. [37]. Field-level correlations between GV and intercept would occur in situations with low winds and higher build-up of water vapor whereas strong correlations between GV and slope would occur if consistent, moderate winds created advection of moisture across fields. A higher concentration of water vapor would be confirmed though a positive correlation with water vapor slope if winds are consistent and moderate, or an increase in intercept if winds are faint and/or variable.

Fourth, this study evaluated Hypothesis F by evaluating the slopes and intercepts of the fitted water vapor surfaces over fields of different irrigated crop species. These intercepts indicate the magnitude of water vapor above a field while the slope is indicative of the trend of vapor over a field. A one-way ANOVA was performed to assess differences in slopes between the crop species, and results were evaluated based on published water demand by species. Crop water use was calculated using the crop ET coefficient for irrigated crops for June in the Southern San Joaquin Valley of California in a dry year [38].

To further examine expected patterns of water vapor as it relates to ET while controlling for some level of complexity within the scene, we chose three crops that are prevalent in the study area and looked at their LST as it related to water vapor slope. To test Hypothesis G, we computed water vapor over fields of alfalfa, almonds, and cherries. We included all fields which had a fractional green cover of 50% or more.

## Results

**Fig 5:**
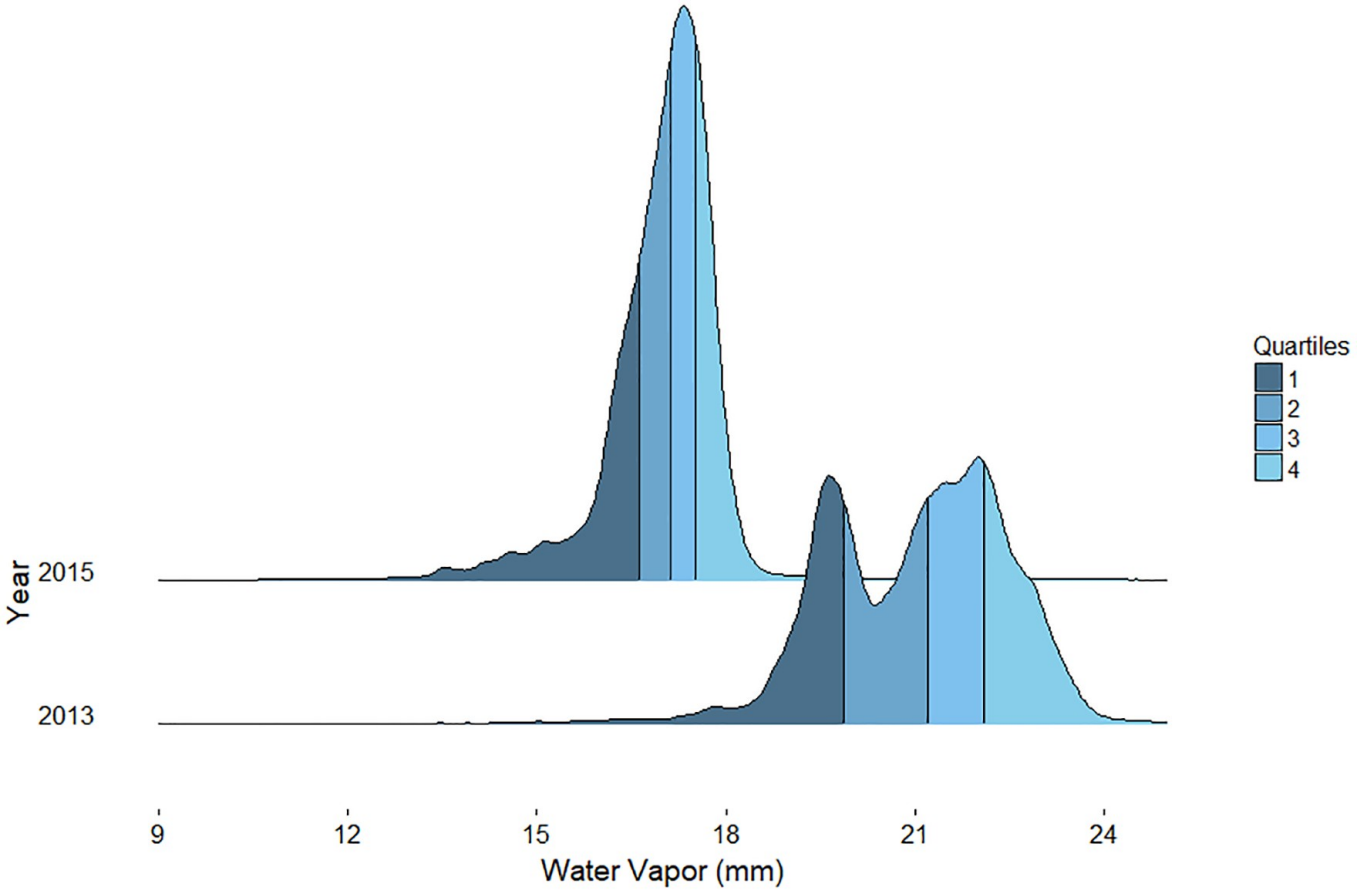
Water vapor distributions by year.

Water vapor concentrations, calculated using all pixels in a flight line, varied significantly between dates with mean values of 21.1 mm, and 17.0 mm in 2013, and 2015 respectively (Fig 5). While 2015 had a normal distribution, 2013 showed a bimodal distribution. The image from 2015 had the least variance in water vapor values with a standard deviation of 0.99 mm as compared to 1.46 mm in 2013.

Calculating linear surface trends of water vapor over fields, in 2013, 84% of the 2,591 fields had statistically significant water vapor surface trends, and 92% of the 3,186 fields in 2015 did. The average slopes from 2013 and 2015 were 0.86 and 0.70 μm of water vapor per meter of field respectively.

### Scene-scale spatial trends in water vapor concentrations

**Fig 6:**
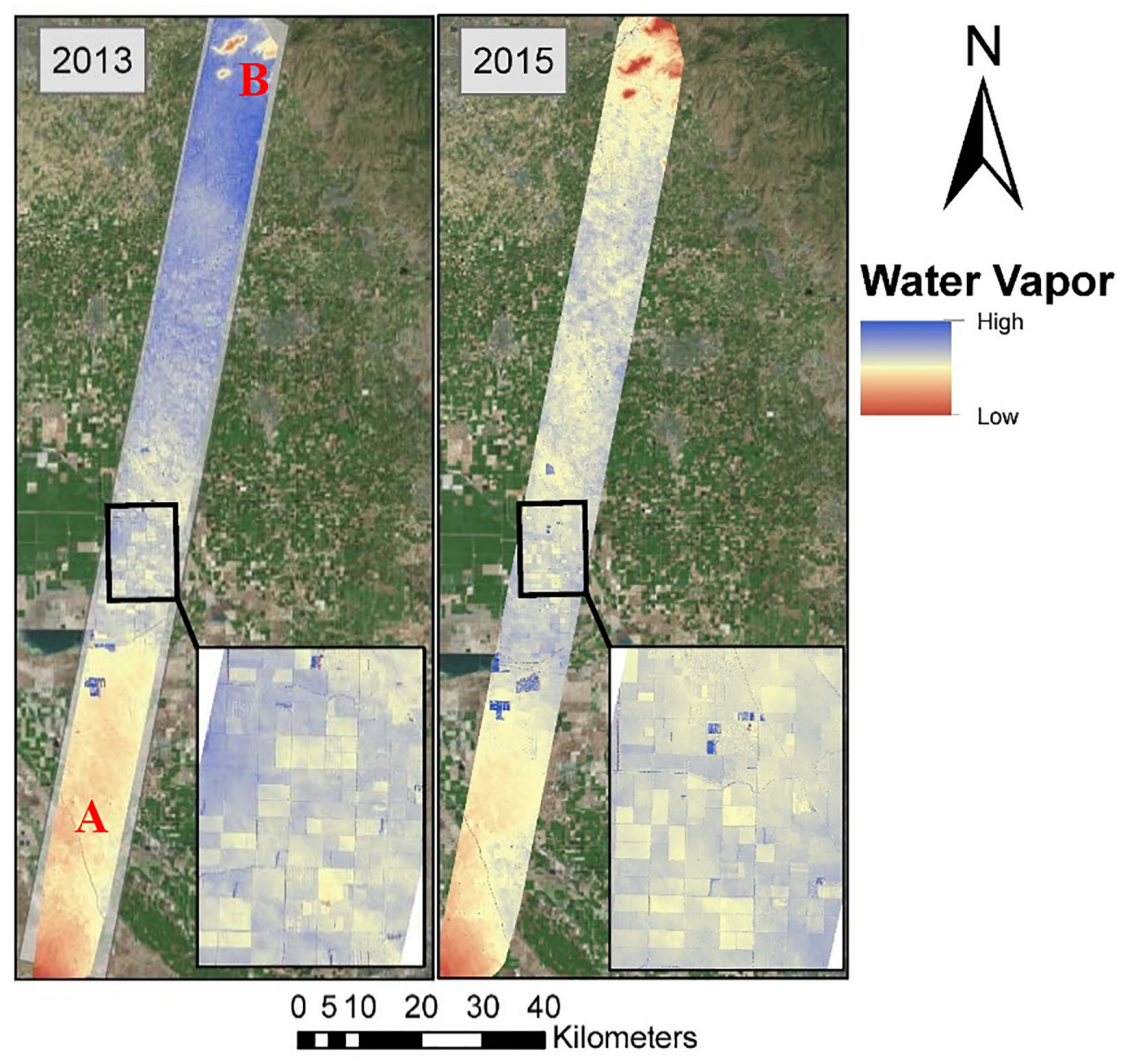
Water vapor images produced using AVIRIS radiance imagery and ACORN reflectance retrieval algorithm. The area between A and B corresponds to the low relief, densely cropped portion of the flight line. Basemap obtained from Esri ArcGIS. Sources: Esri, DigitalGlobe, GeoEye, i-cubed, USDA FSA, USGS, AEX, Getmapping, Aerogrid, IGN, IGP, swisstopo, and the GIS User Community.

The two dates of imagery showed slightly different spatial trends of water vapor. In 2013, water vapor showed a clear increasing trend from southwest to northeast. This trend was significant enough that it outweighed the modest elevational trend, in which elevation increases from 58 m in the central portion of the flight line, to 114 m in the northern portion. This increase in elevation, which translates to a decrease in the distance between the sensor and ground, should result in a decrease in column water vapor [16]. This trend was also present in 2015, but was not as pronounced. Four areas in the northern part of the flight line, with low intercepts in both dates (B on Fig 6) are sparsely vegetated hills, with elevations ranging between 510 m (Campbell Mountain) to 600 m (Jesse Morrow Mountain). Very low water vapor in the farthest south portion of the flight lines is also likely a product of surface elevation, which rises to 900 m. However, in densely vegetated portions of the flight line (regions between A and B on Fig 6), elevations only range between a low of 53 to a high of 120 m.

When observing the imagery at a larger scale, the water vapor from 2013 and 2015 shows strong coupling with the ground surface below with agricultural field boundaries clearly defined. This result is consistent with surface-atmosphere interactions with individual fields.

**Fig 7:**
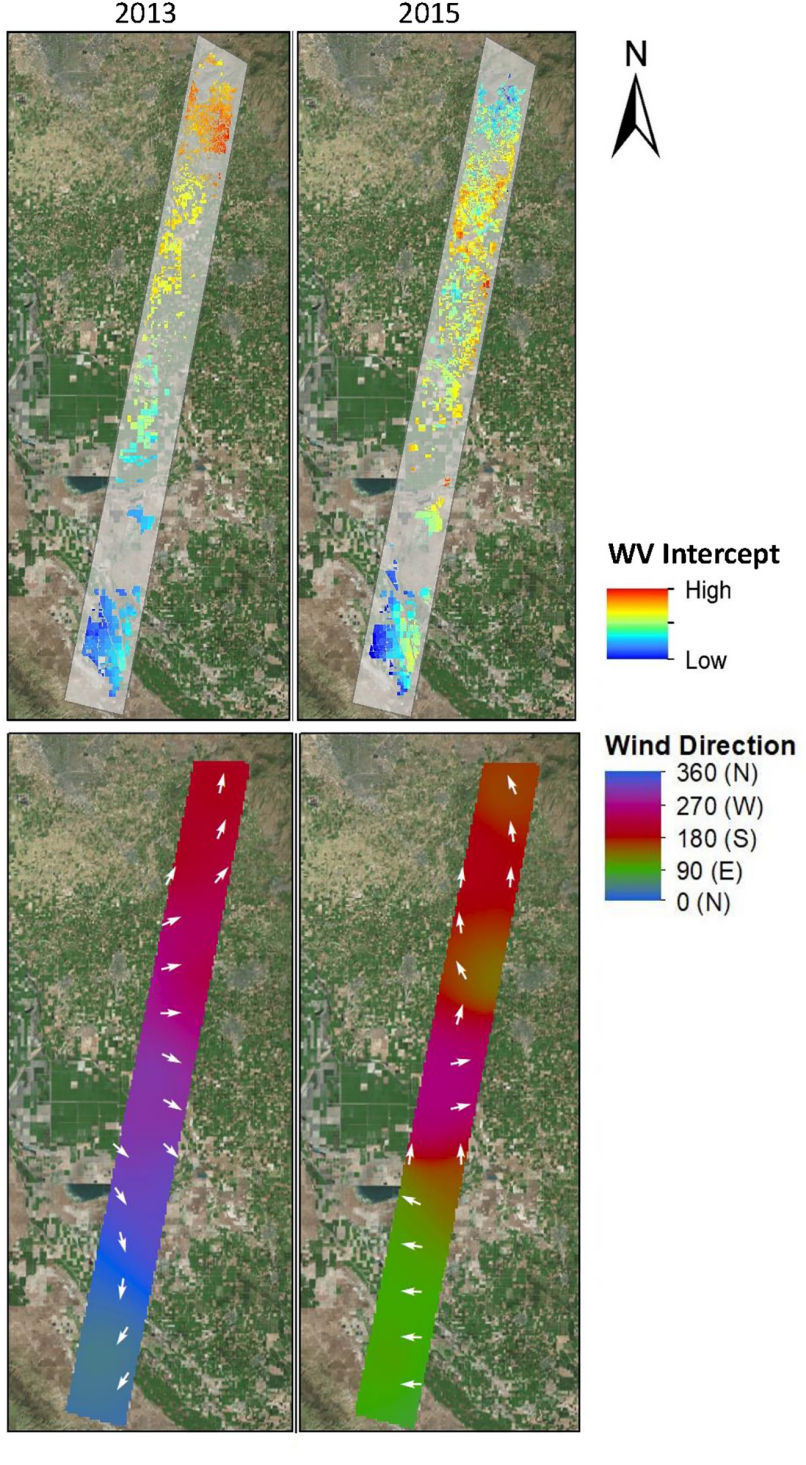
Water vapor intercepts and wind direction maps. Field-level intercepts calculated from water vapor regressions are shown above time-of-flight interpolated wind direction maps for 2013and 2015. Basemap obtained from Esri ArcGIS. Sources: Esri, DigitalGlobe, GeoEye, i-cubed, USDA FSA, USGS, AEX, Getmapping, Aerogrid, IGN, IGP, swisstopo, and the GIS User Community.

Computation of water vapor intercepts and interpolation of wind directionality allowed for comparison between water vapor abundance and patterns of wind as laid out in Hypothesis A. Fig 7 shows the directionality of the wind and the water vapor intercept maps side-by-side for comparison. The 2013 imagery shows the most clear pattern of advected moisture that generally agrees with the wind map, especially in the northern portion of the study area. The intercept map shows water vapor concentration increasing from south to north while the wind direction map shows a south to north trend of wind in the northern part of the study area. As crops transpire and water vapor advects, the intercepts above fields should show increasing moisture. The southern portion of the study area shows less agreement with winds, indicating winds coming from the northeast but a water vapor gradient increasing from west to east. The 2015 image shows water vapor that is not as clear in its trends. The 2015 water vapor intercepts show patterns that are somewhat similar to 2013 with a general south to north increase in moisture, except for in the most northern portion of the flight line. In principle, there should be a relationship between wind direction and a field-based intercept. Progressing downwind, the intercept over a field will be the product of moisture from that field and any additional moisture added to the atmosphere from fields located upwind, as was found by Ogunjemiyo et al. [4]. This appears to be the case in 2013, but is not as strongly evident in 2015. In part, this may be a product of the modest elevational gradients present along the flight line. However, it also may be a product or poor representation of winds at the field scale. The wind map is a snapshot at the time of flight, while the intercept is the product of ET and wind-driven advection that are not instantaneous.

### Field-level water vapor trends

#### Water vapor patterns as a function of wind

**Fig 8:**
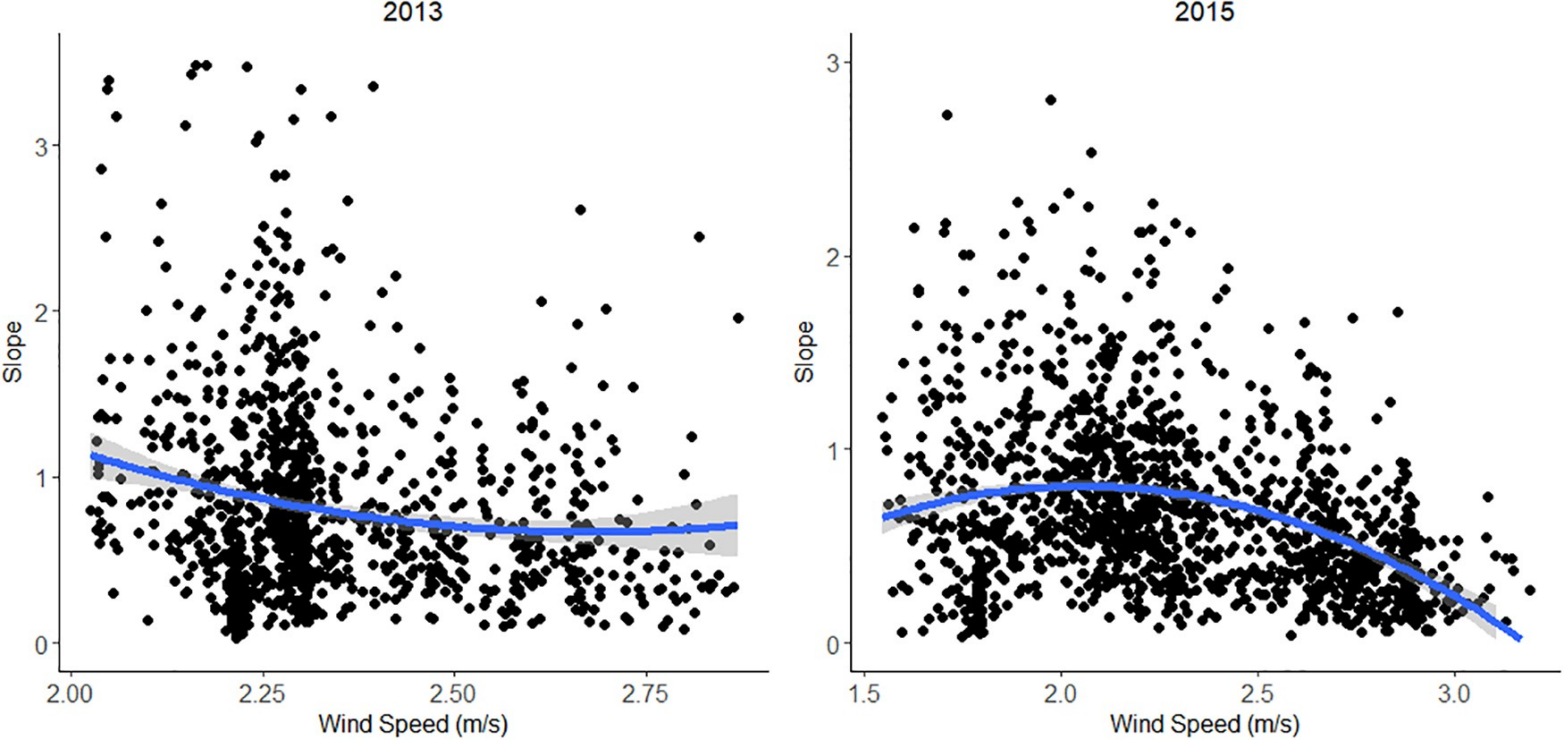
Slope of water vapor and the wind speed above the field show significant quadratic relationships for 2013 and 2015. 2013:Slope = 11.73–8.41*WS+1.60*WS^2^, r^2^ = 0.03, p<0.001, n = 1130; 2015:Slope = -2.20+2.78*WS-0.67*WS^2^, r^2^ = 0.05, p<0.001, n = 1442.

Hypotheses B and C anticipated relationships between the directionality of water vapor and its slope with both wind magnitude and wind direction. When calculating field-scale trends, 84% of the fields in 2013 showed statistically significant water vapor surface trends, and 92% of fields showed significant trends in 2015, indicating that water vapor gradients were detectable in the imagery. When examining fields that were predominantly covered in green vegetation (GV>0.5), we found patterns that showed relatively high variability but were consistent with our hypotheses that a moderate wind speed would show higher slopes than very low wind speeds or high wind speeds (Hypothesis C). This was particularly true for 2015, which had the highest range in wind speeds, highest r^2^ (0.05) and a clear peak near 2 m s^–1^. Although r-squared values were low, both years showed significant quadratic relationships between water vapor slope and wind magnitude (Fig 8).

**Fig 9:**
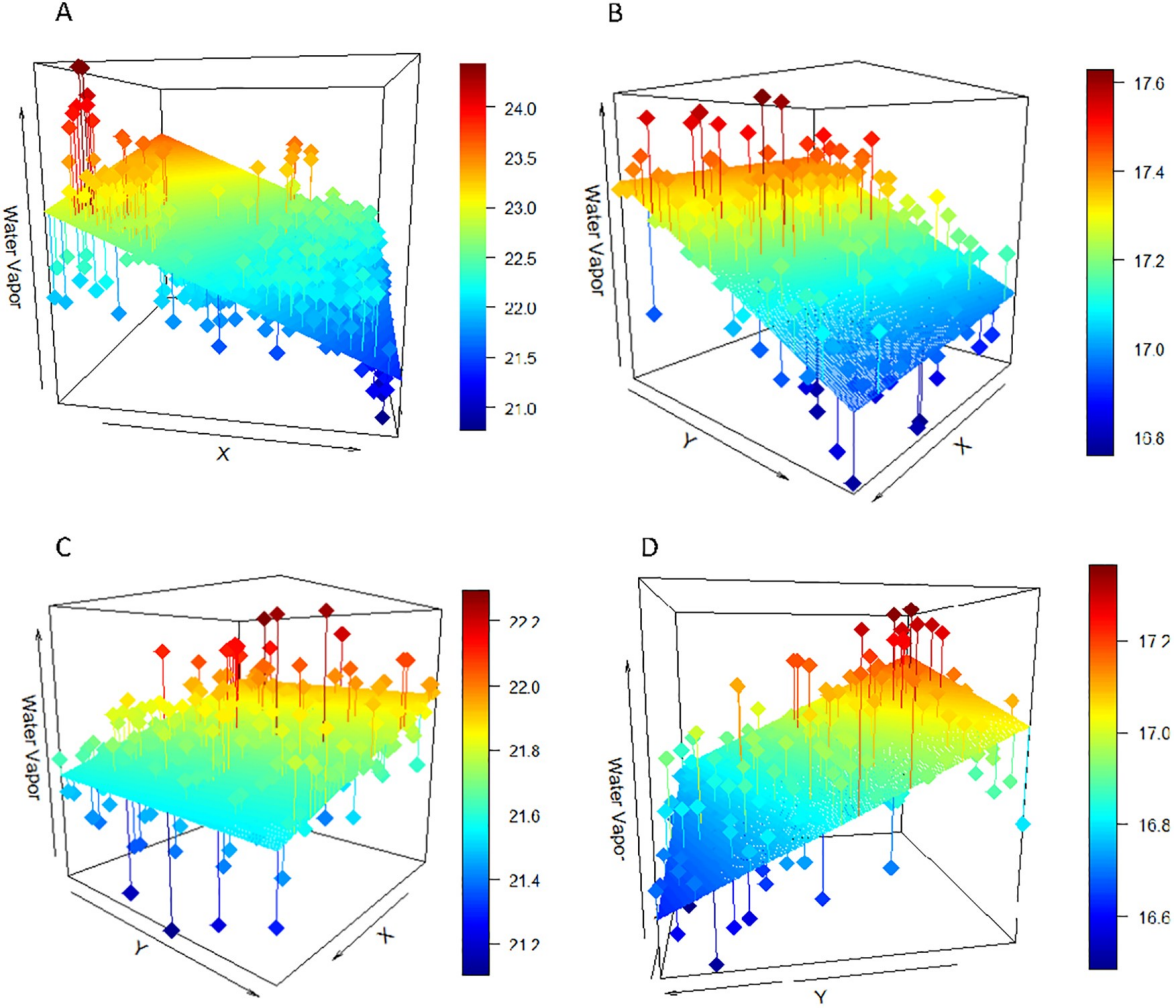
Linear surface trends for four fields. A: Corn field from 2013 (slope: 2.45, r^2^: 0.54, directional difference: 20°), B: Pistachio field from 2013 (slope: 0.55, r^2^: 0.34, directional difference: 7.7C: Peach field from 2015 (slope: 1.18, r^2^: 0.55, directional difference: 7.0°), D: Mandarin field from 2015 (slope: 1.40, r^2^: 0.59, directional difference: 3.8°).

We also hypothesized that wind direction and water vapor trajectory would align if fields were actively transpiring (Hypothesis B). Sub-selecting fields that met our 30° degree criteria for alignment only 787 fields out of 5779 (5107 with significant trends) met the criteria. Of those, 275 were in 2013 and 512 in 2015, which accounted for roughly 11% and 16% of fields in those years respectively. Fig 9 highlights two fields from each 2013 and 2015 that showed vapor patterns that were consistent with wind direction, as hypothesized.

Analyzing all directionally aligned fields by GV cover and crop type in each year, we found no significant characteristics related to GV when these sub-selected fields were compared to all fields in the study. When examining histograms of GV fraction within the fields that showed directional agreement, no discernable pattern was found. High GV fields were as likely to align in trajectory with wind direction as were low GV fields. In fact, the mean GV of the selected fields was 0.45and 0.43 for 2013 and 2015, in comparison to 0.47 for the average of all fields in the study. In contrast, grouping the fields by crop type showed significant differences in the proportion of fields in which the water vapor slope was aligned with wind direction (Table 3). Almost a quarter of the pistachio and almond fields showed water vapor trends that were in agreement with the wind direction while walnut, cherry and grape fields had roughly half that amount. These differences may be attributable to differences in the crops themselves such as rate of ET or health of the plants, or these differences could be attributable to their position within the study scene as the crops are not evenly disbursed throughout the area.

**Table 3:** The most prevalent crops in the study area and their proportion of fields that agreed with wind direction (<30° difference).

Crop	Fields that show agreement with wind direction
Pistachio	22%
Almond	22%
Alfalfa	16%
Peach	15%
Orange	14%
Plum	13%
Walnut	11%
Cherry	10%
Grape	8%

#### Water vapor gradients by field size

Hypothesis D predicted that water vapor gradients would increase with field size. In both years, we found a significant relationship between field size and water vapor slope. However, we also found that field size and water vapor slow were inversely correlated, with correlations of –0.37, and –0.28 respectively. Counter to our expectation, smaller fields generally showed steeper slopes than did larger fields.

#### Water vapor gradients and concentrations by GV fraction

**Fig 10:**
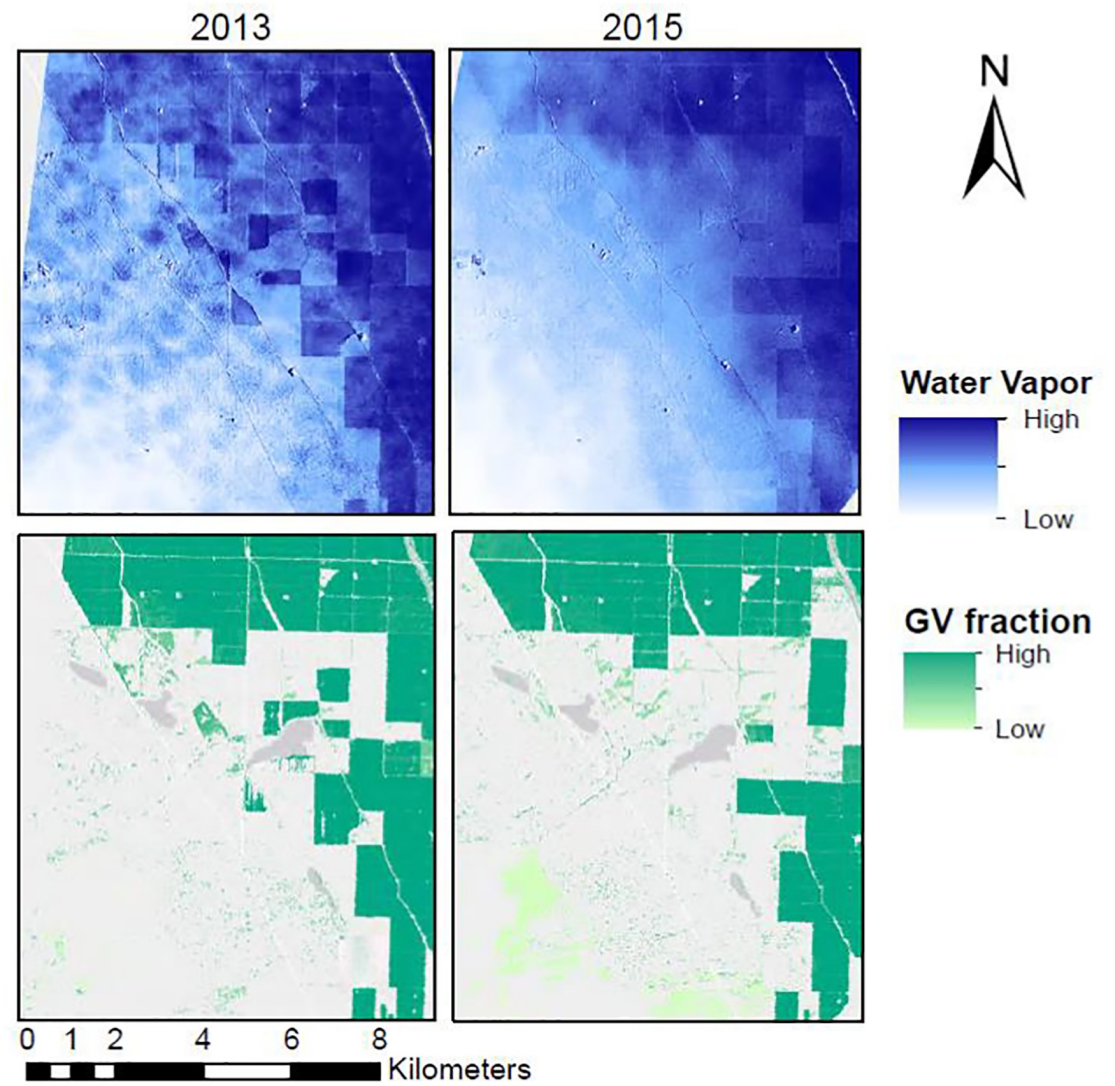
Subset of the water vapor and GV fraction in the southern study area. Water vapor imagery and GV fraction results from 2013, and 2015 illustrate how water vapor occurs over both cropped and fallow areas in part of the study area (35.463°, -119.696°). 2013 and 2015 images show greater concentrations of water vapor over cropped than non-cropped areas. The location of the subset is shown on Fig 6 marked with an A. Basemap obtained from Esri ArcGIS. Sources: Esri, DeLorme, HERE, MapmyIndia.

We hypothesized that GV fraction would positively correlate with slope and/or intercept, dependent on the wind speed (Hypothesis E). Fig 10 shows one example from the southern portion of the study area in a transition zone between non-cropped and cropped areas, where water vapor concentrations showed a large positive gradient from non-cropped to cropped areas as expected.

**Fig 11:**
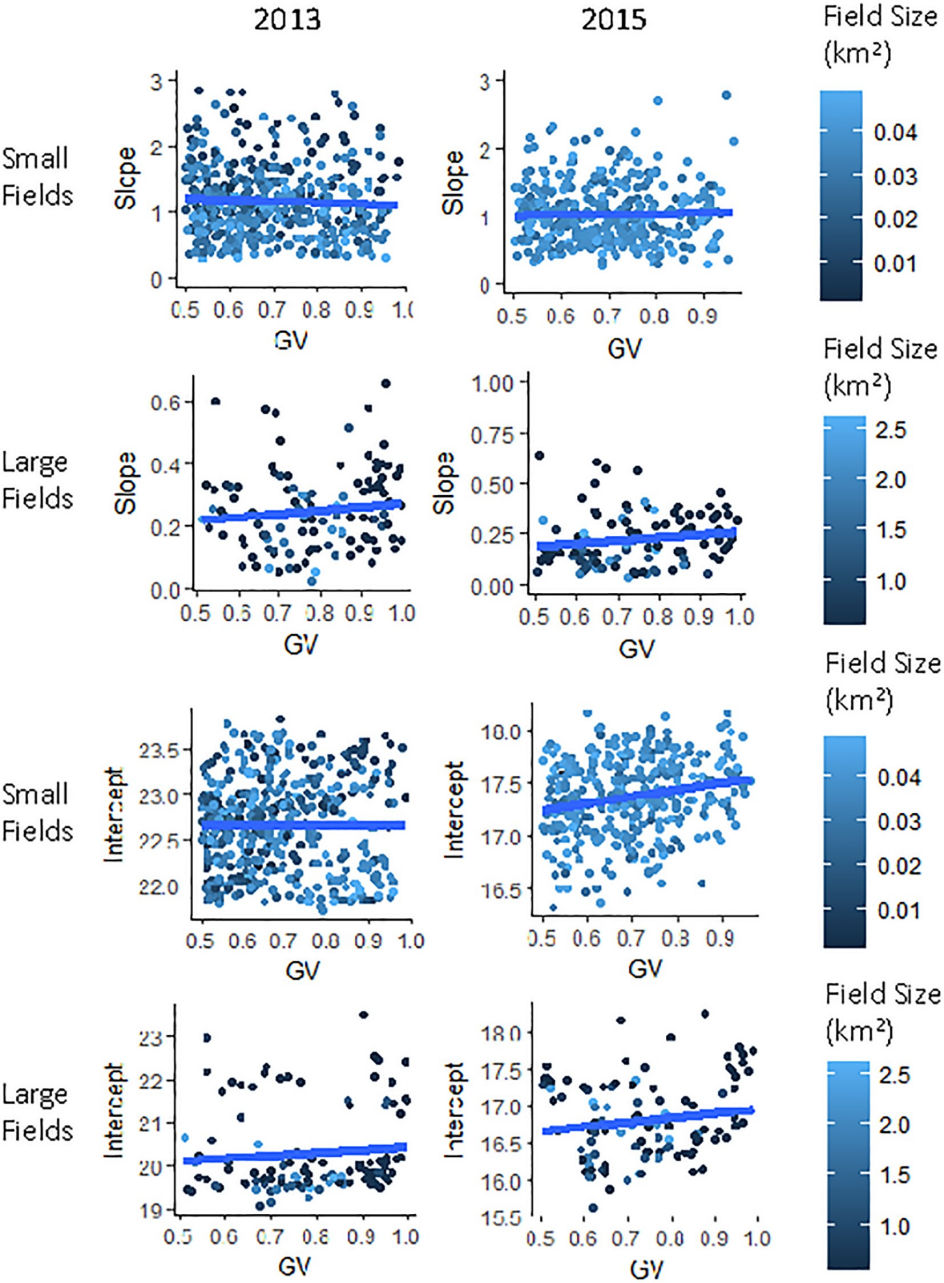
GV fraction by water vapor slope and intercept. Fields from 2013 and 2015, disaggregated by size, show no relationship between GV fraction and slope of water vapor (p>0.05). Small fields from 2015 showed a significant positive relationship between intercept and GV fraction while small fields in 2013 and all large fields showed no significant relationship (p>0.05).

However, quantitative evaluation of field-level water vapor trends within the whole study area, did not find a consistent relationship between GV fraction and water vapor slope (r = 0.001 in 2013, r = 0.02 in 2015) and only a weak positive correlation between GV fraction and intercept (r = 0.11 in 2013, r = 0.26 in 2015). In hypothesis E, we hypothesized that the slope/GV correlation would be depend on GV fraction as fields that are bare or sparse will show no gradients. Splitting the fields into groups of greater than 50% GV and less than 50% GV, we again found no correlations between GV and water vapor slope with all correlation coefficients equaling less than 0.12 and greater than -0.12. Because we found that field size and slope correlated, we further disaggregated fields by field size to study the GV/slope relationship. Fig 11 shows the smallest (less than 0.5 km^2^) and largest fields (greater than 0.75 km^2^) as example groups of the GV/slope and GV/intercept relationships when split into groups of similar size. No significant trends were found between GV and slope. Small fields from 2015 showed a significant positive linear relationship between GV and intercept (2015: 16.9 + 0.65*GV, r^2^ = 0.04, p<0.001) while the small fields in 2013 and large fields both years showed no significant relationship (p>0.05).

#### Water vapor by crop species

**Fig 12:**
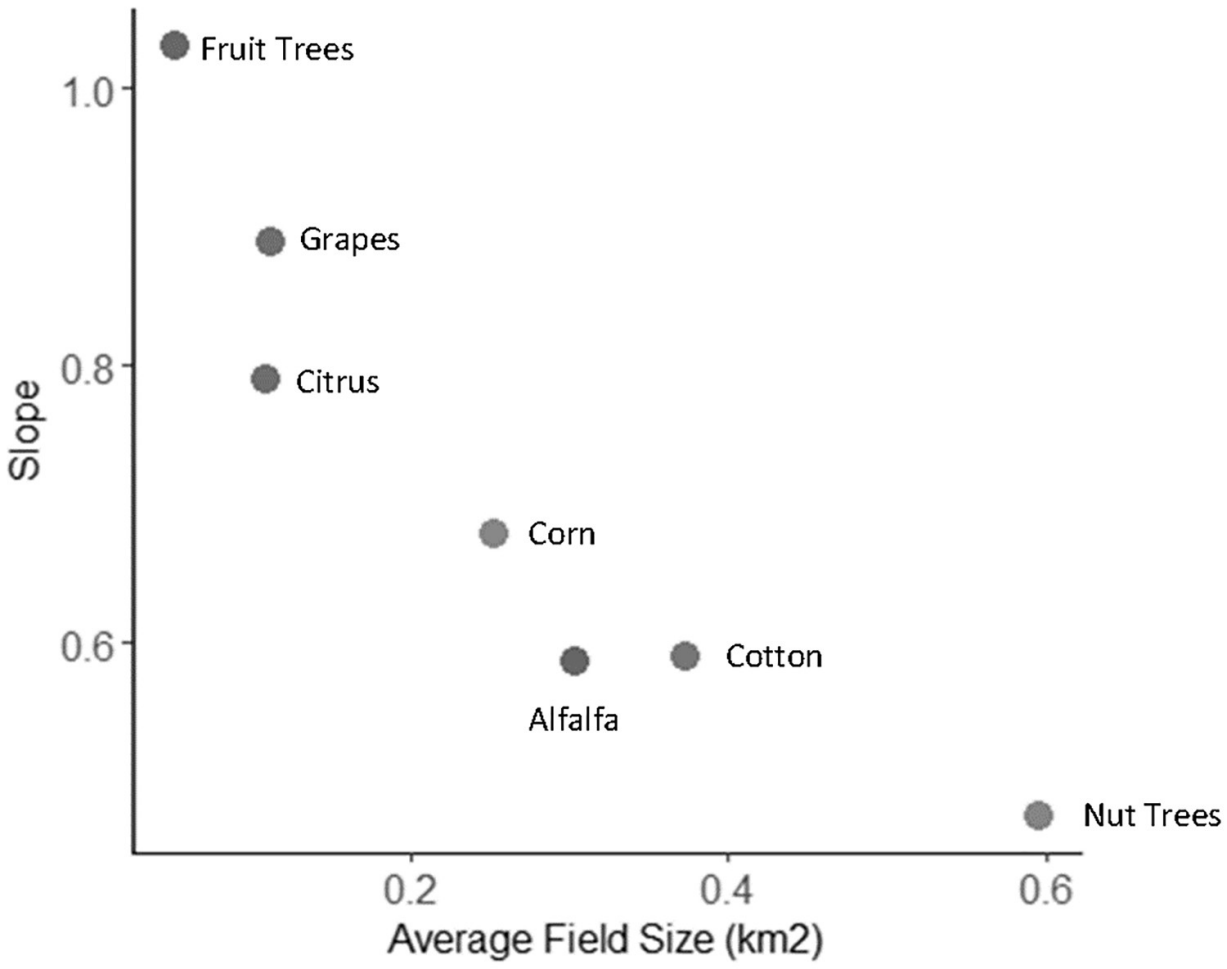
Water vapor slope by field size. Plot showing a negative relationship between water vapor slope and average field size in 2013 for seven crop types prevalent in the study area.

A one-way ANOVA showed that crops do have significantly different slopes of water vapor in both years (2013: [F(6, 1760) = 33.6, p < 0.001], 2015: [F(6, 2490) = 39.6, p < 0.001]). We assessed the average slope of each crop type with its expected water use to test Hypothesis F, and expected to find a positive correlation with crops that have higher water use also having higher slopes of water vapor above them. However, we did not find a significant linear trend when plotting expected water use against average slope either year (p>0.05). Given the results of the field size study, we tested the relationship between average slope and average field size and found a significant negative linear relationship in each year (2013: Slope = 0.96–0.94*Size, p<0.001; 2015: Slope = 0.90–1.34*Size, p<0.01). Fig 12 shows 2013 as an example. Therefore, the differences in water vapor slope by crop type, as found by the one-way ANOVA, may be attributable to the average field size of these crops instead of their water use as hypothesized.

**Fig 13:**
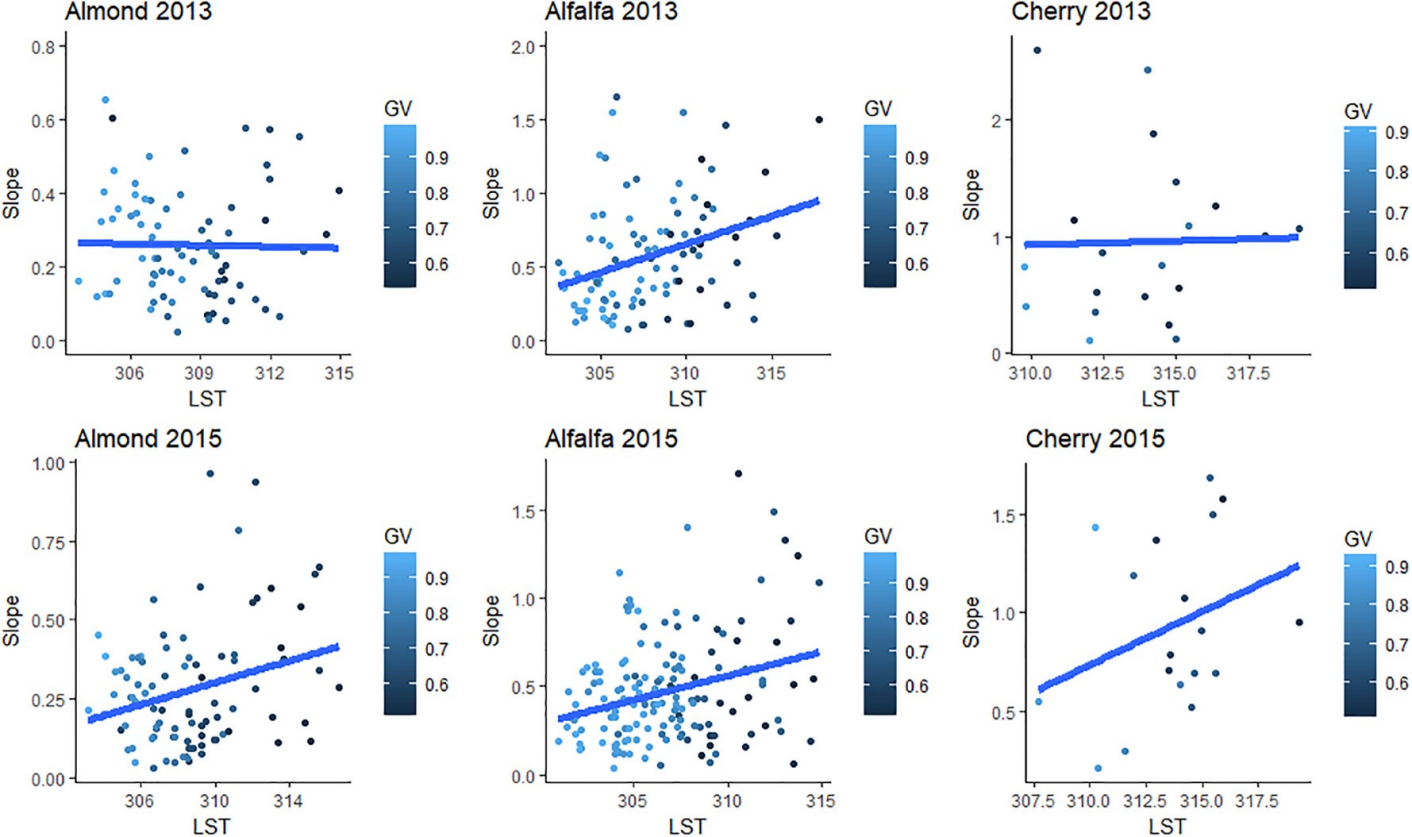
Field-level values of water vapor slope and field-level LST for alfalfa, almond, and cherry from 2013 and 2015. Points are colored by fractional GV coverage. Alfalfa 2013: Slope = -10.0+ 0.03*LST, p = 0.013; Almond 2013: Slope = -6.6 + 0.02*LST, p = 0.074; Cherry 2013: Slope = -1.1 + 0.007*LST, p = 0.914; Alfalfa 2015: Slope = -7.9 + 0.03*LST, p<0.001; Almond 2015: Slope = -5.0 + 0.02*LST, p = 0.008; Cherry 2015: Slope = -16.2 + 0.05*LST, p = 0.195.

Examining patterns of water vapor over fields within a single crop species, we plotted LST, which we use here as an indicator of plant water stress or reduced transpiration rate, against water vapor slope for fields of alfalfa, almonds, and cherries (Fig 13). As would be expected for actively transpiring crops, LST was lowest in the fields where GV fraction was highest. However, we also expected to find a negative relationship between LST and water vapor slope as the cooler fields should have higher rates of evapotranspiration leading to an increase in water vapor above a field, detectable through slopes (Hypothesis G). However, in contrast to Hypothesis F and G, LST and water vapor slope showed a positive correlation that was significant for alfalfa in both years and almonds in 2015 (Fig 13). This finding suggests that fields with more healthy, green vegetation have lower slopes, on average, than less vegetated or stressed fields. The reasons for this finding will be explored further in the discussion.

## Discussion

Water vapor imagery contains information regarding the land surface, the atmosphere, and their interactions, and thus offers a valuable dataset with which to observe and quantify key fluxes between the two. In this study, we expanded upon the work of Ogunjemiyo et al. [4] by testing several hypotheses to investigate how AVIRIS estimates of water vapor vary with the surface properties and atmospheric conditions in the Central Valley of California. In this section, the key findings will be interpreted, the challenges of this methodology will be discussed, and thoughts on how to move this work forward will be presented.

### Interpretation of results

The results supported that water vapor imagery will show coupling with the land surface at the pixel-level and advection at the scene-scale, under certain atmospheric conditions (A). Further, results supported hypotheses that water vapor gradients will form over fields as a function of wind speed and direction (B-C). Crops with higher water demand showed higher agreement between the water vapor trajectory and interpolated wind direction but not water vapor slope (F). Hypotheses that water vapor slope will increase with field size (D), would correlate with green vegetation fraction (E) and would be higher for lower LST crops were not supported.

By calculating field-level water vapor intercepts and evaluating them over the scene, we found evidence that supported Hypothesis A, particularly in the 2013 scene. In that image, small-scale trends of vapor across the study scene showed patterns that were consistent with advection of moisture, as shown in Fig 7. This finding suggests that, as air moves across the Central Valley, crop ET adds water vapor to the atmospheric column, which builds up in the downwind direction and is detectable in regional scale imagery. The presence of a regional gradient in 2013 that runs counter to an expected decrease in water vapor with increasing elevation is particularly compelling.

Hypotheses B and C were supported by significant quadratic relationships between wind magnitude and slope that suggest that water vapor slopes only occur when the wind is strong enough to create such trends but weak enough that the water vapor slope is not too shallow. Work by Ogunjemiyo et al. [4] found their conceptual model held best when winds were at 1.17 to 1.24 ms^-1^. The one case that showed no water vapor patterns was with an August image with 3.91 m/s winds. The winds in our study were between 1.5 and 3 m/s, in between Ogunjemiyo’s values of 1.24 to 3.91 ms^-1^. The quadratic result of our wind speed vs. slope curves suggest that relationships were strongest at intermediate speeds from 2.2 to 2.5 ms^-1^, and that the wind speed in Ogunjemiyo were lighter than their optimal speed for creating water vapor slopes. However, there are also significant differences between the two sites, including high levels of irrigation and high levels of ET in the clonal *Populus* studied by Ogunjemiyo [4].

**Fig 14:**
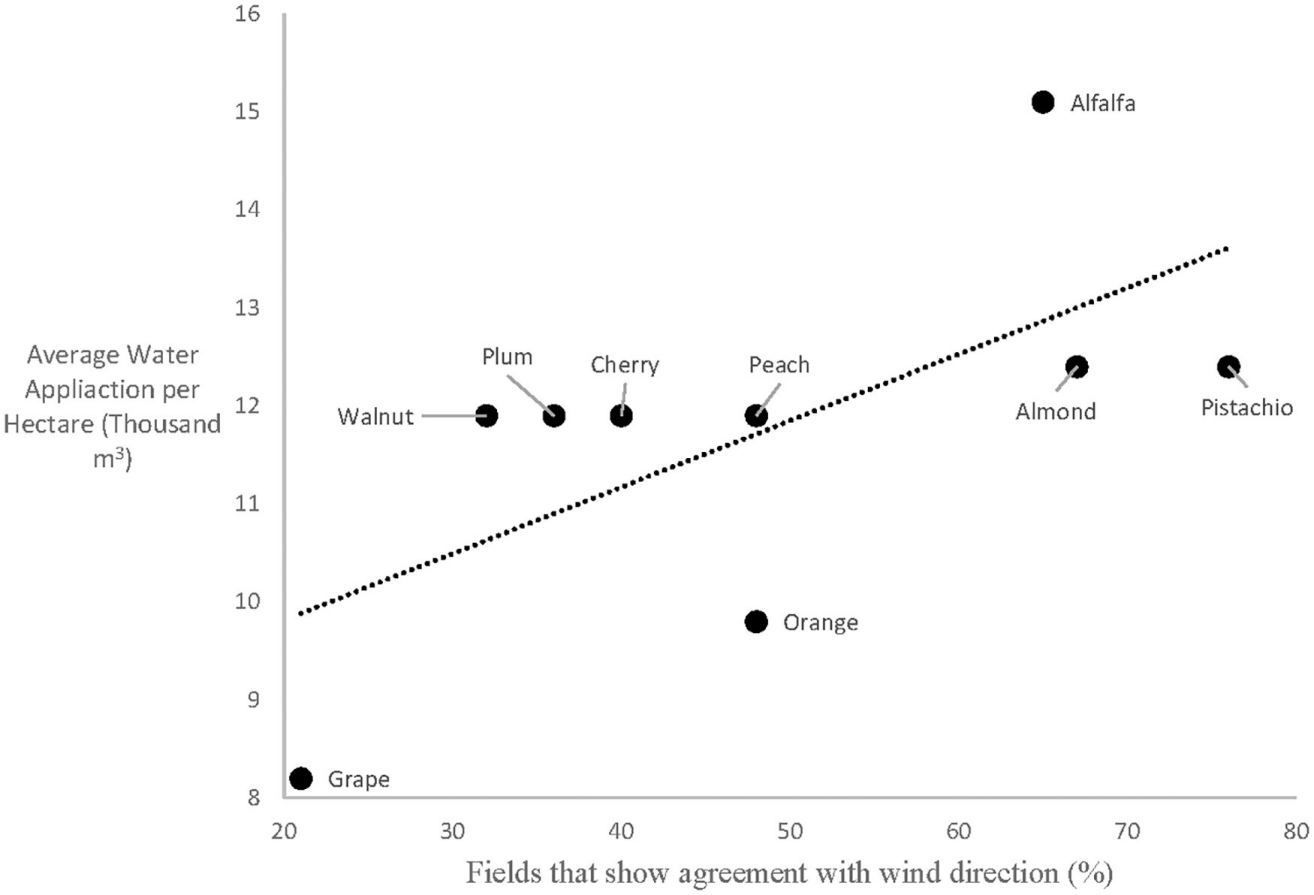
Water vapor trends by crop water use. A positive correlation is found between crops that require higher water inputs and those that show better agreement between water vapor trends and wind direction (y = 0.02x + 8.87).

Some fields showed water vapor trends consistent with our hypotheses regarding water vapor trajectory and wind direction, supporting Hypothesis B. Within pure GV pixels there existed a large range of temperatures that suggests that not all green crop fields were transpiring, possibly due to water shortages mid-summer during a severe drought. Therefore, we hypothesize that fields that show directional agreement between water vapor patterns and wind could be indicative of those fields that were actively transpiring at the time of flight. In agreement with this hypothesis, we found the proportion of fields that showed aligned with interpolated wind direction varied by crop type (Table 3). The three crop types that had the highest rates of water application, namely alfalfa, pistachio and almond, also showed the highest proportion of fields in which water vapor slope aligned with wind direction (Fig 14] Plotting the percentages in Table 3 with the water application values of each crop from Shivers et al. [37], we found a positive correlation (r = 0.23; Fig 14). This result suggests that high water-use crops more frequently show water vapor patterns that align with the wind direction, which is consistent with inputs of crop transpiration the local column water vapor.

Hypotheses E, F and G were not supported. These hypotheses all emanated from a larger idea that water vapor slope would correlate with ET. We did find that water vapor slope correlated with field size (Hypothesis D), but the relationship was negative, with larger fields showing lower water vapor slopes. One possible explanation is that the signal of water vapor movement becomes more diffuse and less concentrated over larger fields due to variation in wind speed and direction. For small fields, the length of time required to move a parcel of air from one end of the field to the other will be low, and thus the likelihood that the wind speed and direction will be consistent will be higher, resulting in a more pronounced gradient. As field size increases, the length of time required to move a packet of air from one side of the field to the other will increase, decreasing the probability that wind speed and direction will remain relatively constant. Furthermore, as the moisture content increases down wind, this would decrease vapor pressure deficit, potentially reducing rates of ET downwind. Another explanation, suggested by that fact that some crops showed a positive correlation between LST and slope, is that rather than advection of plant-transpired moisture downwind over individual fields, there is instead an accumulation of water vapor over the field. This idea will be explored further in Section 4.1.3.

Second, we did not find positive correlations between GV fraction and water vapor slope as postulated in Hypothesis E. If green vegetation is transpiring and adding to the water vapor above a field, we would expect higher fractions of GV to contribute more water vapor, and thus increase the size of the gradient. We found no correlation between water vapor slope and the GV fraction, even when results were segmented by field size and GV fraction. We used 50% GV as the cutoff to demarcate sparsely vegetated fields from highly vegetated fields, as is consistent with previous studies [37]. However, we found that the average fractional GV coverage of fields that showed good alignment between wind direction and water vapor directionality was around 45%. Therefore, future studies may want to consider a lower GV threshold or a segmentation of fields into multiple GV classes.

Finally, we did not find an inverse correlation between water vapor slope and LST in support of Hypothesis G. Either no correlation was found, or highest water vapor slopes were found with higher temperature crops.

#### New hypotheses

Water vapor patterns were as expected at the field level, in response to wind. However, water vapor patterns were not as expected in response to the surface properties of field size, GV fraction, and ET rate as expressed by field-scale LST. We had hypothesized that field-level water vapor slopes can be used to infer crop transpiration, but did not find evidence supporting that hypothesis. Rather, our results suggested that water vapor accumulation from transpiration was more dominant than the advection signal at the field level. The rate of ET has been found to remain constant with downwind distance across a field, even if warm, dry air is being advected toward a vegetated field [39,40]. If plants are transpiring at a constant rate and winds are not strong enough or stable enough in directionality to evenly advect the moisture, the concentration of water vapor above the field would increase relatively evenly throughout the field, leading to a diminished slope. Crops are also more aerodynamically rough than an empty soil field [41], and the resultant turbulence caused by vegetation creates eddies and atmospheric mixing that may muddle signals of field-level advection discernable above smoother landscapes. The hypothesis of water vapor accumulation is supported by results that found a positive relationship between LST and slope for some crops, a negative relationship between field size and slope, and a weak positive correlation between water vapor intercept and GV fraction in 2013 and 2015. Therefore, the results of this study lead us to new conceptual understanding that the magnitude of water vapor as assessed though the intercept of a fitted plane may be better indicator of ET than the slope. However, underlying heterogeneity of the landscape and scaling issues, as discussed below, prohibited isolated analyses of intercepts in this study area.

### Challenges of water vapor analysis

Observing the link between water vapor and the land surface is complicated by multiple factors that obscure the signal. These include error within the water vapor and wind estimations, the heterogeneity of the landscape, and spatiotemporal issues of scale.

#### Estimation error

There is error within all water vapor estimates regardless of which retrieval method is used, and the estimates vary significantly from model to model [42]. However, Ben-Dor et al. [42] found that, of six different water vapor retrievals, ACORN estimated water content with acceptable accuracy and, importantly for our study, it was one of only two models that accurately discriminated water vapor from liquid water in plants. Therefore, the positive correlations found in years 2013 and 2015 between water vapor and vegetation fraction are assumed to be a product of coupling between the landscape and the atmosphere, rather than an artifact of the retrieval.

Poor knowledge of winds at the field scale also represent a significant limitation. Wind direction and magnitude can change significantly within a small period of time, making estimations of wind within the study scene at the time of the flight particularly difficult. Furthermore, a sparse network of meteorological stations, may not accurately capture more local variation in wind between the stations. Thus the IDW wind field we used in this study may not adequately characterize fine spatial or temporal variability in winds at the field scale.

#### Land surface interactions

**Fig 15:**
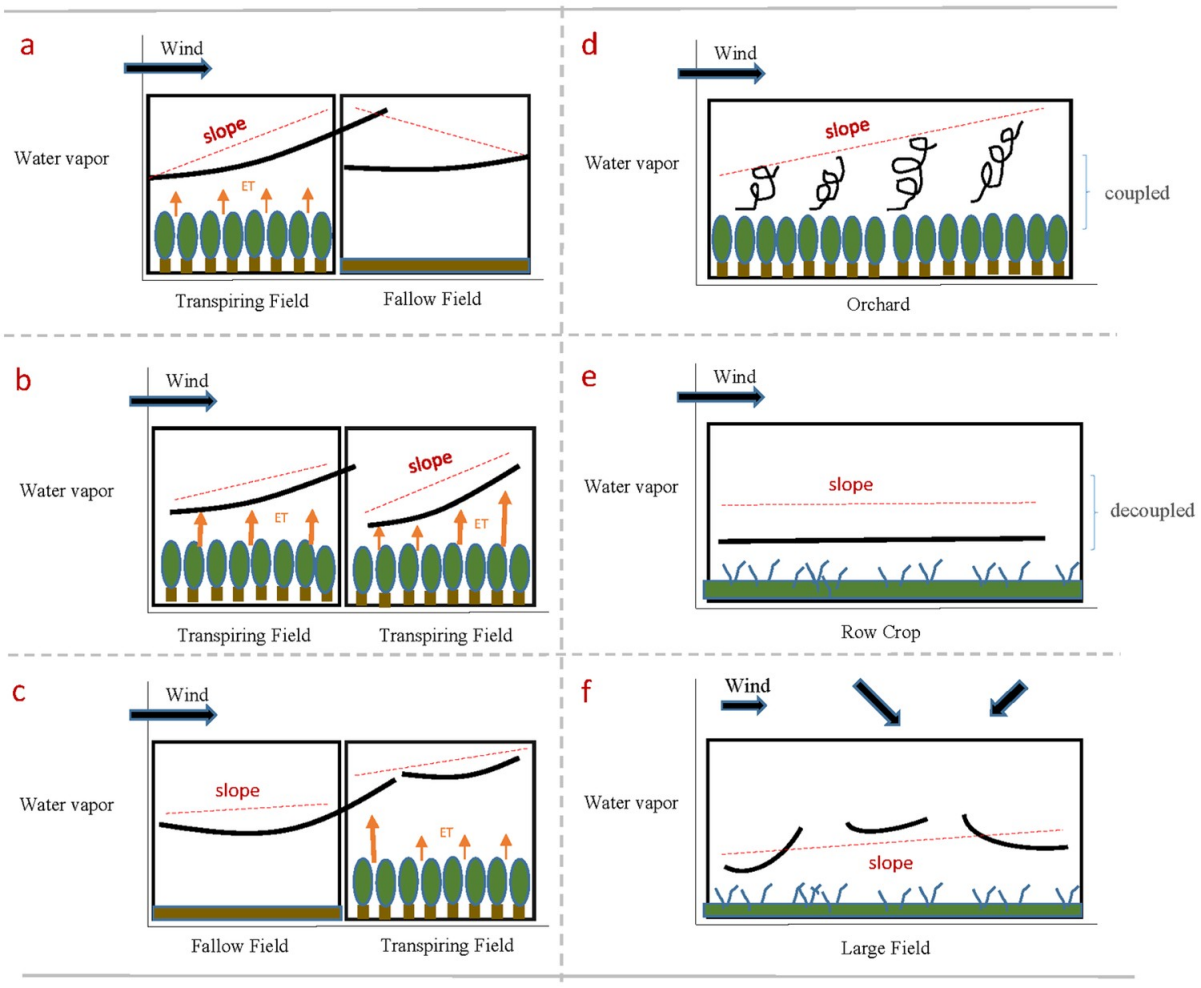
Field and canopy-level factors affecting water vapor trends. Schematic of water vapor over agricultural fields as it is affected by adjacent fields (A,B,C), as it changes with field roughness (D,E) and as it changes with wind heterogeneity (F). Black lines represent water vapor while red dotted lines indicate estimated slopes.

Unlike Ogunjemiyo et al. [4] who studied water vapor over a relatively homogeneous area of transpiring poplar trees, this study evaluated water vapor as it varies across a very diverse agricultural landscape with many different crop species, green vegetation cover, and irrigation regimes. As such, Ogunjemiyo’s conceptual model (Fig 1) illustrated an ideal relationship between water vapor and vegetation at the field-scale that may not hold in our complex study area. First, interactions between water vapor occurring over two diverse, adjacent fields may alter the vapor deficit and stomatal response of a single crop field and result in water vapor trends that do not follow Ogunjemiyo’s model. The schematic in Fig 15A illustrates one possible interaction in which a transpiring field is upwind of a non-transpiring field. While the transpiring field will act as hypothesized with the slope and direction of a fitted plane in line with the wind direction, a plane fitted to the fallow field downwind will likely show a slope that is opposite in direction to the wind. The wind carries moist air from the vegetated field onto the fallow field, leading the upwind edge of the fallow field to have higher water vapor concentrations than the edge that is downwind. In the case of the downwind area being another highly transpiring field (Fig 15B), the moist, advected air from the upwind field may reduce the transpiration rate of the downwind field at the boundary by decreasing the vapor pressure deficit. This may lead to an exaggerated water vapor slope over the downwind field. The accumulation of water vapor from one field can therefore lead to shifts in vegetation response that are difficult to account for. Fig 15C illustrates the scenario where a dry, fallow field is upwind of a transpiring field. If the area upwind of a vegetated field is fallow, we would expect the saturation deficit of the dry advecting air to increase the evaporation rate at the boundary unless the vapor pressure deficit is high enough to initialize stomatal closure [40]. A higher ET rate at the upwind side of the field will lessen the expected, observable trend of advection across the field. The transpiration response will be species-dependent.

Second, not all fields will interact with the atmosphere in the same ways, due to differences in aerodynamic roughness, affected by row spacing, plant height, plant size, orientation, and composition. The aerodynamic roughness of a field will influence how effectively and at what height the transpired water vapor will mix with the atmosphere [41]. Agricultural fields may differ strongly in aerodynamic roughness, and these differences will lead to deviations from the hypothesized water vapor slope and intercept patterns as they vary with crop type. Therefore, we would not expect all fields to show the same relationships between water vapor, wind, and estimated transpiration rates. We would expect aerodynamically rougher surfaces, such as orchards, to generate greater turbulence, generate mixing higher up in the atmosphere, and show greater coupling with the wind than row crops (Fig 15D). Depending on the wind speed, orchards may show higher or lower slopes than row crops if their vapor patterns are more tied to wind patterns. In contrast, shorter and smoother row crops such as alfalfa will be less coupled to the atmosphere (Fig 15E). Because crops such as orchards are more closely coupled to the atmosphere, they may be more appropriate to study with water vapor imagery.

Therefore, isolating the effects of neighboring fields would be beneficial for field-level water vapor analyses, but this was not logistically possible in our study. The study area is a high-producing agricultural area where most fields are bordered by multiple neighbors of varying GV cover, crop type, size, physical characteristics that influence roughness, and ET rate. Further, without LiDAR data from which physical characteristics such as orientation, height and structure could be obtained, it was not possible to model field-scale differences in aerodynamic roughness in this study. This work has aimed to enhance understanding of the impact of GV fraction, field size, crop type and water use on patterns of water vapor. Positive findings include the presence of significant vapor gradients over most fields, and regional patterns in water vapor that are consistent with advection. High water use crops also showed a disproportionally higher level of agreement between interpolated wind direction and the direction of water vapor gradients. Field size impacted water vapor slope, although slopes were higher in smaller fields than larger fields, in contrast to expectations. We suspect improved knowledge of winds at the field scale, would improve our ability to interpret water vapor gradients. For example, given that a majority of the fields showed statistically significant water vapor slopes, an alternative hypothesis may be that those gradients better represent winds at the field scale, than interpolated winds from a sparse network of stations. Finally, we found the intercept of the best-fit surface for water vapor over a field to be more significant than the slope, suggesting that water vapor is accumulating over fields, rather than advecting.

### Opportunities for future work

Water vapor imagery shows patterns of vapor that are highly variable through space and time and that hold valuable information about land-atmosphere interactions. We suggest there is considerable potential for this imagery and explored some of this potential here.

To further scientific understanding of water vapor imagery analysis, further studies are necessary to refine observation and quantification of land-surface interactions as the signal is highly complex and is affected by many factors. While water vapor imagery could potentially be used to parameterize models of land-surface interactions, additional studies in a diversity of landscapes are necessary to define the conditions and scales at which this imagery can be used. Almost 4,000 AVIRIS images have been collected since 2006 and are available for public download. With such a large repository of data collected at different time points, under varied atmospheric conditions, and over diverse surfaces, future research could tease out the conditions under which interactions can best be observed in a more comprehensive way than this study of three snapshots in time could. Further, with future remote sensing missions such as SBG, which will collect hyperspectral imagery at moderate spatial resolutions and enable column water vapor estimates globally, these data streams can be exploited for comparisons of water vapor over large agricultural areas worldwide. These large archives of water vapor observations can also act as a compliment to models that estimate water vapor and plant water use by providing validation data.

In addition to increasing analysis of similarly complex scenes, future studies would benefit from additional data sources that could isolate the signal of water vapor and validate its link to the surface. Such controls include on-site continuous wind measurements, flux tower measurements of ET, and/or more spatially comprehensive wind data. On-site wind data and ET measurements at a high temporal resolution would both validate trends seen in the water vapor imagery and assist in pinpointing the appropriate temporal scale and time of day for which this analysis is best suited. A mesoscale weather model such as the Weather Research and Forecasting Model (WRF), might also provide a more accurate fine scale representation of wind fields than simple IDW of weather stations as used here. A finer network of weather stations, and or controlled experiments with meteorological equipment deployed in advance of a flight at specific fields would also be of benefit.

Although more work is needed in order to refine understanding of the water vapor signal in a complex agricultural environment, the results suggest that this technique could be of use for crop water analyses in agricultural areas that experience less variation in crop type, wind, and field size than the Central Valley of California. As an example, Ogunjemiyo et al [4] studied a more simplified crop setting, consisting of a single, highly evapotranspiring crop in an area with simplified meteorological conditions (more uniform prevailing winds and a more arid natural vegetation). Similar uniformity occurs throughout much of the Midwest, where mono-cultures of corn and soybean would permit the study of water vapor patterns while reducing the impact of variation in crop type. The Central Valley, with its large variety of crops and management practices, resulting in non-uniform distributions of aerodynamic roughness, ET rates, and landscape structures throughout the scene, was perhaps not the ideal location to test this approach. Unfortunately, the type of data we used, including AVIRIS–derived water vapor, and LST from MASTER, is not widely available outside of data sets acquired for the HyspIRI-Airborne Campaign. We would suggest a targeted campaign, acquiring combined AVIRIS-MASTER for agricultural studies, over more simplified and better instrumented sites would be of great benefit.

Beyond advancing our ability to capture patterns of field-level ET with water vapor imagery, this imagery may prove valuable for regional analyses of water transport. There are many challenges associated with linking water vapor to crops at the field-level as outlined above, but the idea behind this work will likely hold at a smaller scale. Lo and Famiglietti [13] found that in the Western United States the irrigation from California has been shown to increase the summer streamflow of the Colorado River by 30 percent. Water vapor imagery, if acquired more consistently and over larger areas, offers an additional tool that could be used to capture finer scale water vapor transport, to complement models and coarser scale observations from sensors such as MODIS. These large movements of water vapor have implications for climate change and land use, and call upon the need to increase monitoring of water vapor patterns in areas with large irrigation inputs. Therefore, a study that examines the ability of water vapor imagery to assist in regional water transport assessments could be of high value.

## Conclusion

This paper analyzed water vapor as it varies spatially in two years over a complex agricultural landscape. We tested a series of hypotheses primarily focusing on advected moisture at field and regional scales, assessing patterns through a linear fit of water vapor surfaces at the field scale. We found strong evidence for regional scale advection from croplands. At the field scale, we found significant quadratic relationships between water vapor slope and wind speed with highest slopes observed at intermediate wind speeds. Crop water use, and the frequency in which crops showed directional agreement between wind direction and water vapor trajectories were positively correlated. However, contrary to our hypotheses regarding the impact of field size and green vegetation fraction on water vapor slope, water vapor slope was inversely correlated with field size and showed no statistically significant relationship to vegetation fractional cover. Water vapor slope was also positively correlated with field-scale LST, contrary to an expectation that higher rates of evapotranspiration (and lower leaf temperatures) would resutl in higher water vapor slopes. We propose an alternate hypothesis that evapotranspired moisture accumulates, rather than advects over most of these fields, as expressed in the intercept of the best fit plane. We suggest that the existing archive of AVIRIS-derived water vapor imagery has considerable potential, but additional research is needed to better define how evapotranspiration is expressed in water vapor images. A targeted field campaign, combining an imaging spectrometer and thermal sensor, with field-scale instrumentation is warranted.
